# Feasibility and Cognitive Benefits of a Computerized Training Program in Dementia Prevention in Portugal

**DOI:** 10.1192/j.eurpsy.2026.11574

**Published:** 2026-07-31

**Authors:** A. Martins, B. Lopes, C. Gameiro, P. Varandas, C. Pombo, M. V. D. S. Nunes

**Affiliations:** 1 Aging and Dementia, Sisters Hospitallers; 2 Faculty of Nursing and Health Sciences, Center for Interdisciplinary research in Health (CIIS), Universidade Católica Portuguesa, Lisboa, Portugal

## Abstract

**Introduction:**

Multidomain interventions for dementia prevention have shown consistent benefits and have been standardized through the FINGERS model, with the cognitive dimension being particularly relevant. Computerized cognitive training is increasingly used, but its feasibility in Portugal – where many older adults have low educational levels and limited digital literacy – remains underexplored. This study analyses the cognitive component of DemenPrev, a Portuguese feasibility study adopting the FINGER model.

**Objectives:**

To evaluate (1) adherence to the computerized cognitive training program; (2) usability and satisfaction; (3) progression across cognitive domains; and (4) differences between initial and final platform assessments.

**Methods:**

Twenty older adults with the mean age of 71.9 (SD=3.6) at risk for dementia participated. Seventeen participants were women (85%), and three were man (15%). The mean years of education were 9.7 (SD=4.0). Baseline assessment was conducted using three computerized exercises: Mind Bender (executive functions), Target Tracker and Double Decision (attention). Based on these results, individualized training plans were generated. Participants attended two 45–50 minute sessions per week over 12 weeks. Post-intervention, the same exercises were repeated for reassessment.

**Results:**

Adherence was very high (95.2%), and participants expressed strong willingness to continue. Significant improvements were observed in executive functions (Mind Mender, p=0.006) and attention (Target Tracker, p=0.004; Double Decision, p<0.001). Satisfaction parameters (motivation, engagement, demand, time, clarity) were rated from moderately to extremely satisfactory. Usability was highly rated by the group. (M=71.63).

**Conclusions:**

Preliminary findings suggest that computerized cognitive training can improve executive functions and attention, with excellent adherence and high satisfaction among Portuguese older adults. While causality cannot be established without randomized controlled trials, these results support its potential as a promising non-pharmacological component of dementia prevention strategies.

**Disclosure of Interest:**

None Declared

